# The Incidental Diagnosis of Adenomatoid Tumour on Fallopian Tubes Submitted for Tubal Ligation at a Tertiary Laboratory in Northern Pretoria, South Africa

**DOI:** 10.1177/2632010X241230265

**Published:** 2024-02-16

**Authors:** Nosipho Maria Thobakgale, Moshawa Calvin Khaba

**Affiliations:** 1 Department of Anatomical Pathology, Dr George Mukhari Tertiary Laboratory, National Health Laboratory Service, South Africa; 2 Sefako Makgatho Health Sciences University, Pretoria, Gauteng, South Africa

**Keywords:** Fallopian tubes, adenomatoid tumour, tubal ligation, incidental

## Abstract

**Introduction:**

Adenomatoid tumours are the most common benign mesothelial neoplasms of the fallopian tube. They are usually diagnosed incidentally in specimens submitted for bilateral tubal ligation and can be mistake for vascular or epithelial lesions.

**Materials and methods:**

A retrospective analysis of cases with adenomatoid tumour of the fallopian submitted for tubal ligation from 2012 to 2020. The clinicopathological characteristic data was retrieved from the laboratory information system.

**Results:**

A total of 11 cases with adenomatoid tumour of the fallopian tubes submitted for tubal ligation were identified in women with average age of 30.9 years. In all the cases, only 1 fallopian tube was affected. Grossly, the fallopian tubes did not show any discernible tumour. Immunohistochemical stains confirmed the diagnosis of adenomatoid tumours in all the cases.

**Conclusion:**

Adenomatoid tumours in fallopian tubes are infrequent, and pathologists shouldn’t overlook them especially in unsuspicious instances. As frequent as adenomatoid tumour of the fallopian tubes are uncommon, pathologists show be aware of as their misdiagnosis could lead mismanagement of patient with far reaching complication.

## Introduction

Adenomatoid tumour (AT) is a rare benign tumour that can affect women of any age, although it is more prevalent in women of childbearing age. It commonly involves the uterus and rarely fallopian tubes and ovary; however, it is the most common benign neoplasm in the fallopian tube.^1-4^

The hypotheses regarding the histogenesis of these tumours, such as endothelial, mesonephric, mullerian and mesothelial origin have been discussed. However, the morphological, immunohistochemical and ultrastructural features supports the mesothelial origin.

The majority of patients have no unusual clinical symptoms and signs hence the difficulty in preoperative diagnosis. Majority of these cases are incidental findings while treating patients for other illnesses.

Most of the cases are diagnosed on microscopic examination of tissues submitted for other conditions, such as uterine leiomyoma, ovarian carcinoma, tubal ligation and tubo-ovarian abscess.^5^

In this study, we discuss 11 cases of adenomatoid tumours of the fallopian tube that were diagnosed incidentally on tissues submitted for bilateral tubal ligation. To the best of our knowledge, this might be the first series reported from South Africa.

## Materials and Methods

This study was conducted in accordance with the regulations and with the approval by the Sefako Makgatho Health Sciences University research ethics committee, SMUREC/M/99/2023, Institutional Review Board, IRB000010386.

All biopsies that were diagnosed and coded as ‘adenomatoid tumour’ in the fallopian tubes submitted for tubal ligation from 01 January 2012, to 31 December 2022, were accessed from the archive of the Department of Anatomical Pathology, Dr George Mukhari Academic Laboratory, National Health Laboratory Service, Ga-Rankuwa, Gauteng, South Africa, using the Systematized Nomenclature of Medicine Clinical Terms (SNOMED) code and word search engines. Departmental records of these patients were accessed, where available, to record clinical details such as age, sex, race and site of biopsy.

Stored hematoxylin and eosin–stained sections were reappraised by 2 pathologists (MCK and NMT). Where necessary, archive formalin fixed paraffin embedded (FFPE) blocks were sectioned at 4-μm thickness for hematoxylin and eosin staining.

In-house modification of internationally recognized protocols with appropriate positive and negative controls were used for the immunohistochemical (Table 1). Archived FFPE blocks were sectioned at 3-μm thickness for the spectrum of immunohistochemical stains.

**Table 1.** Antibodies.

Antibody/probe	Clone	Dilution	Retrieval	Control	Supplier
WT-1	6F-H2	RTU	High pH target retrieval	Kidney	Dako
Anti human podoplanin (D2-40)	D240	1:50	High pH target retrieval	Appendix	Dako
Calretinin	DAK-calret 1	RTU	High pH target retrieval	Appendix and adrenal gland	Dako
CD34	Q Bend 10	RTU	High pH target retrieval	Liver and appendix	Dako
CD31	JC70A	RTU	High pH target retrieval	Tonsil and liver	Dako
Anti human cytokeratin	AE1/3	RTU	High pH target retrieval	Skin	Dako

Abbreviation: RTU, ready to use.

## Results

A total of 11 cases met the inclusion criteria and formed the study cohort.

### Clinical features

The study consisted of 11 females with mean age of 30.9 years (range, 27-40 years). All the cases were submitted for tubal ligation as part of family planning.

Of the 11 cases, only 1 was described as being distended intraoperatively.

### Histopathologic features

#### Gross features

Of the 11 patients, only 1 had a dilated fallopian; the others appeared normal.

#### Histomorphologic features

**Figure 1.**
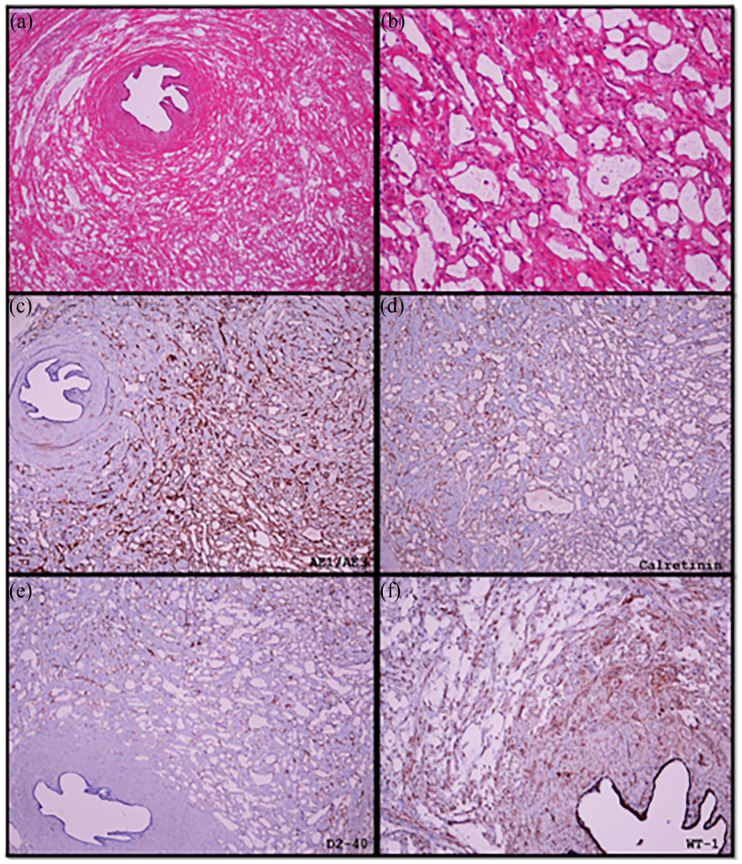
Adenomatoid tumour of the fallopian tube. (a) Normal fallopian tube lumen with tumour involving the muscle. (b) Cystic spaces lined by single layer of cuboidal cells. (c) Positive for pankeratin marker (AE1/3). (d), (e) and (f) Positive calretinin, D2-40 and WT-1, respectively.

In each case, adenomatoid tumour was noted in the fallopian tubes as an uncapsulated and circumscribed tumour arranged in tubular formations with intervening fibromuscular stroma. Cuboidal cells with eosinophilic and vacuolated cytoplasm lined the tubules. The nuclei had fine chromatin with conspicuous nucleoli (Figure 1a and b). Cytological atypia, mitosis or necrosis was not seen. There was only 1 fallopian tube affected by AT in each of these cases.

#### Immunohistochemical features

All the 11 cases showed strong and diffuse immunopositivity for WT-1, D2-40 and calretinin, which is indicative of mesothelial origin. D2-40 and calretinin stained the cytoplasm and nucleus while WT-1 stained the nucleus (Figure 1d-f). AE1/AE3 was also strong and diffusely positive. CD34 and CD31 immunostains were negative in all the cases, ruling out a vascular origin.

## Discussion

Adenomatoid tumours were originally termed benign mesothelioma by Masson et al in 1942.^6^ The term Adenomatoid tumour was first introduced by Golden and Ash in 1945^7^ to describe benign, incidental mesothelial tumours of the genital tract.^5^ The term was adopted by Ragins and Crane^8^ 2 years later, in 1947 to denote tumours that were previously diagnosed as adenomas of the fallopian tubes.^8^

Adenomatoid tumours are benign neoplasms of mesothelial origin commonly involves both female and male genitourinary system.^3^ Extragenital involvement is extremely rare, this include cases from the adrenal gland, peritoneum, liver, pleura and mediastinum.^5^ In the female genital tract, uterus is commonly involved and fallopian tube is rarely involved.^1^ With regards to male genital tract, epididymis and testicular tunics are commonly involved.^5^

Four major hypotheses have been postulated regarding the histogenesis of adenomatoid tumours over the years including endothelial, mesonephric, mesothelial and mullerian epithelium origin.^1,9^ Masson et al. proposed mesothelial origin of these tumours for the first time in 1942, using macroscopic and microscopic finding of serosal surface origin and electron microscopy findings.^10,11^ The theory was proven by Stephenson and Mills in 1986 by using electron microscopy, mucin histochemical and immunohistochemical features.^9^

Adenomatoid tumours of the female genital tract are usually discovered incidentally.^3,4^ There are few cases were patients presents with clinical diagnosis of leiomyoma, endometriosis, adenomyosis and ovarian mass.^5^ In this study, all the cases of AT were incidental findings; however, in 1 case a distended fallopian tube was identified intraoperatively which was suggestive of a mass. Withstanding this, adenomatoid tumour was not considered in the latter.

Grossly, adenomatoid tumour in the fallopian tube are small, circumscribed and solitary which measures less than 2 cm in diameter^1^; in contrast to tumours of the uterus and male genital tract that can show an infiltrative growth pattern.^5^

Microscopically, there are several histopathological patterns identified which may include cystic, solid, adenoid, papillary and angiomatoid pattern.^3,11^ The cells are bland with moderate to scant eosinophilic cytoplasm. The nuclei are round to oval with fine chromatin and small conspicuous nucleoli.^4^ The cells may have eosinophilic and vacuolated cytoplasm. Atypical cells (in areas of infarction) and signet ring cells have been described which may be confused with malignant processes.

Additional features may include lymphoid aggregates and psammoma bodies^2,5^ In this study, all the cases showed adenoid patterns with vacuolated cell being the predominant cell.

When these tumours undergo infarction with subsequent necrosis and proliferation of reactive fibroblast, they may be mistaken for malignant process.

The differential diagnosis includes benign and malignant lesions. The most common benign lesions in the fallopian tube are lymphangiomas and salpingitis isthmica nodosa. Lymphangioma resembles cystic spaces of adenomatoid tumour; however, the spaces in lymphangiomas contain lymphocytes and stains negative for calretinin despite being positive for D2-40. Salpingitis isthmica nodosa is an outpouching of the fallopian tube epithelium into the smooth muscle wall resembling the glandular pattern of adenomatoid tumour. The glands of isthmica nodosa are lined by tubal-type epithelium. The glands are negative for mesothelial markers.^4^

The common malignant tumours comprise well-differentiated liposarcoma, metastatic adenocarcinoma including signet ring carcinoma and malignant mesothelioma. Metastatic carcinoma often has a multifocal nature, characterized by an infiltrative growth pattern and the presence of surrounding stromal desmoplasia. Mucin production is commonly observed in certain tumours, particularly signet ring carcinoma, but adenomatoid tumours typically do not exhibit this characteristic. Although both tumours exhibit strong expression of epithelial markers, adenocarcinomas do not show any expression of mesothelial markers. Malignant mesothelioma exhibits an infiltrative growth pattern characterized by pronounced nuclear pleomorphism and an elevation in mitotic activity. Mesothelioma exhibits HMBE1 positivity, whereas adenomatoid tumours do not. Additionally, the presence of BAP1 deletion is a valuable diagnostic confirmation tool for mesothelioma.^4,5,8^

Electron microscopy (EM) has been useful in the diagnosis of AT and show long microvilli, tonofilaments, desmosomes and few micropinocytic vesicles.^9,10^ Molecular alteration of this tumour shows somatic missense mutation in TRAF7 gene that drives aberrant NF-Kb pathway activation.^2^ Whilst these ancillary study are important in the diagnosis of adenomatoid tumour, the microscopic features and immunohistochemical stains are enough to reach a diagnosis in most cases as was also evident in this study.

## Conclusion

Adenomatoid tumours are the most common benign neoplasms of the fallopian tubes. They are usually diagnosed as incidental findings in specimens sent for bilateral tubal ligation. However, it is crucial to correctly diagnose these tumours because the various histological patterns and atypical presentations maybe misdiagnosed as malignant neoplasms.
